# Different Models to Predict the Risk of Recurrent Hepatocellular Carcinoma in the Setting of Liver Transplantation

**DOI:** 10.3390/cancers14122973

**Published:** 2022-06-16

**Authors:** Helena Degroote, Anja Geerts, Xavier Verhelst, Hans Van Vlierberghe

**Affiliations:** Department of Gastro-Enterology and Hepatology, Ghent University Hospital, 9000 Ghent, Belgium; anja.geerts@uzgent.be (A.G.); xavier.verhelst@uzgent.be (X.V.); hans.vanvlierberghe@uzgent.be (H.V.V.)

**Keywords:** hepatocellular carcinoma, liver transplantation, recurrence, risk-assessment scoring

## Abstract

**Simple Summary:**

Liver transplantation is considered the first-choice curative therapy for hepatocellular carcinoma in the early phase of the disease, when surgical resection is not possible. Even when implementing restrictive criteria to select patients for liver transplantation, there is a risk of recurrence in the transplanted liver, influencing the long-term outcome and prognosis. As it is challenging to predict the individual risk of recurrence, there is a need for validated and predictive scoring systems to use to stratify patients before and/or after liver transplantation. Most of the proposed scorings include biological markers for tumour behavior, in addition to the number and size of tumoral nodules. In this review, we discuss different published models to assess the risk of recurrent hepatocellular carcinoma after transplantation. Our aim is to refine clinical decisions about prioritization and listing for liver transplantation, to better inform patients and provide an appropriate surveillance strategy to influence their prognosis.

**Abstract:**

Liver transplantation is the preferred therapeutic option for non-resectable hepatocellular carcinoma in early-stage disease. Taking into account the limited number of donor organs, liver transplantation is restricted to candidates with long-term outcomes comparable to benign indications on the waiting list. Introducing the morphometric Milan criteria as the gold standard for transplant eligibility reduced the recurrence rate. Even with strict patient selection, there is a risk of recurrence of between 8 and 20% in the transplanted liver, and this is of even greater importance when using more expanded criteria and downstaging protocols. Currently, it remains challenging to predict the risk of recurrence and the related prognosis for individual patients. In this review, the recurrence-risk-assessment scores proposed in the literature are discussed. Currently there is no consensus on the optimal model or the implications of risk stratification in clinical practice. The most recent scorings include additional biological markers for tumour behavior, such as alfa-foetoprotein, and the response to locoregional therapies, in addition to the number and diameter of tumoral nodules. The refinement of the prediction of recurrence is important to better inform patients, guide decisions about prioritization and listing and implement individualized surveillance strategies. In the future, this might also provide indications for tailored immunosuppressive therapy or inclusion in trials for adjuvant treatment.

## 1. The Burden of Recurrent Hepatocellular Carcinoma after Liver Transplantation

Hepatocellular carcinoma (HCC) is one of the most frequent causes of cancer-related death worldwide, and the disease shows an increasing incidence. Furthermore, HCC is among the leading indications for liver transplantation (LT) globally, and cirrhotic patients with HCC account for 30% to 35% of the population on the waiting list in Europe. LT has the potential to cure tumours and underlying liver disease, which is an important risk factor for the development of new lesions. Because of the limited amount of donor organs, international guidelines offer limited LT to patients, with an assessed long-term outcome comparable to benign indication (with 50% survival at 5 years being the lowest acceptable cut-off). The implementation of selection criteria for transplant eligibility reduced the recurrence rate (RR) [1]. However, even when using strict selection criteria, the risk varies between 8 and 20% of transplantations [2]. This is important, since recurrent HCC after LT generally has an unfavorable outcome, depending on the recurrence characteristics, such as the location and the time period after LT. However, when the recurrence of HCC is detected early and treated aggressively, the outcome of patients can improve dramatically.

The sites of recurrent tumoral lesions after LT are variable and in most cases not limited to the liver. The lungs and bones are the most frequently involved and, to a lesser extent, the adrenal glands, lymph nodes or the peritoneal cavity [3]. A review of the literature showed that in 218 patients, the median time before recurrence was 15.1 months (with a wide range, between 1 and 118 months) [4]. In a cohort study, early recurrence (<12 months) occurred in 32.1% and late recurrence (>12 months) in 67.9% of patients. Liver recurrence appeared early; most lung and bone recurrences occurred later [5]. An observational study of 311 patients with HCC in the explant confirmed a significant decrease in 5-year survival (22%) compared to patients without recurrence (64%) [6]. Regarding the pattern of recurrence, late HCC recurrence, the absence of bone metastasis, low serum AFP levels (<100 ng/mL) at recurrence and the possibility of aggressive treatment were the factors associated with better outcomes [6], with these patients showing a 5-year survival of 50% after diagnosis [7]. A large study stratified the at-risk groups based on the MELD score, donor sodium, neutrophil-to-lymphocyte ratio (NLR), time to recurrence, number and diameter of recurrent nodules, bone location and AFP at recurrence and found a median survival ranging from 70.6 months for the low-risk group and 12.2 months for the medium-risk group to only 3.4 months in the high-risk group [8]. In an Italian case series, the prognosis differed in patients with resectable recurrences (4-year survival: 57%) and unresectable disease (4-year survival: 14%) [9]. In a Latin American multicenter study, treatment with systemic or locoregional therapy ameliorated survival, even in the population with early recurrence and worse outcome [10]. Altogether, the presence of therapeutic options and the characteristics of the recurrence have a significant impact on the survival of patients.

## 2. Use of Milan Criteria to Select Candidates for Liver Transplantation in HCC

In the international guidelines, based on the Milan criteria, LT is recommended for patients with HCC in the early stage of the disease if surgical resection is not suitable. The Milan criteria, published in 1996 by Mazzaferro et al., include a single tumour with a size of 5 cm or less and up to three tumour nodules, each with a maximal diameter of 3 cm, without macrovascular invasion or extrahepatic location [11]. These morphometric variables are clearly established prognostic factors and the restrictive selection of candidates for LT based on the number and size of nodules is used to minimise the risk of recurrence. In the original study, the patients meeting these specific criteria at pathological assessment of the explant showed a recurrence-free survival (RFS) of 92% at 4 years, whereas the rate in the group beyond these limits was significantly decreased to 59% [11]. The predicted 5-year survival rate within these conventional criteria is reported to be between 65% and 78% [1,11,12,13].

Additionally, the Milan criteria are considered the basis for downstaging strategies with locoregional therapies. There is a consensus in the current guidelines that patients with more advanced disease can still be eligible for LT when the tumour burden is successfully downstaged to within the Milan criteria, under the condition that well-defined protocols are used [1]. There are several published studies and meta-analyses on locoregional therapy showing a reduction in the risk of drop-out related to tumour progression. Furthermore, the response to locoregional treatments significantly influences the risk of tumour recurrence after LT and is an indicator for favorable tumour biology [14]. In a systematic review, the success rate of downstaging more advanced HCC to within the Milan criteria exceeded 40% with recurrence rates of 16%. The protocols were reported to be very heterogeneous. The most common practice is to use ablation and/or transarterial therapies, especially when the average waiting time on the list is more than six months [15].

In most of the initial publications, the outcome and risk of recurrence after LT was based on explant findings and then validated in the pretransplant setting, replacing pathology with radiological assessment. In this regard, the Milan criteria were validated in multiple studies [1,11,12,13]. However, a first limitation is the reported discrepancy rate of 20 to 40% between the pretransplant imaging and histopathological staging of liver explants [12]. This resulted from tumour progression during the waiting time, the response to locoregional therapies and/or incorrectly characterised tumour burden on radiological assessment before LT. Secondly, the Milan criteria are considered too strict and some oncological patients with a potentially good prognosis miss the opportunity for curative treatment with transplantation. Recently, the Milan criteria have been increasingly being questioned, since comparable post-transplant outcomes have been achieved with broader criteria.

## 3. Development of Risk-Assessment Models beyond Milan Criteria

The extended criteria are considered to function as pre-transplant recurrence-risk-assessment scores (Table 1, non-exhaustive list). Their key intention is not only to identify patients beyond the Milan criteria without an increased risk of recurrence, but also a high-risk subgroup within the Milan criteria. The use of these extended criteria is included in international guidelines, albeit without further specification or a consensus on their implementation [1,16]. Initially, the expansion of the selection criteria for LT in HCC patients was mainly focused on maximising the acceptable number and diameter of HCC nodules. For example, the extended Asan criteria included patients with a maximum of six nodules, with tumour diameters of up to 5 cm. Patients beyond the Milan but within the Asan criteria showed a 3-year RR of 9.1% in the study. The criteria were validated in Western countries and non-living-donor settings [17,18,19]. The University of California, San Francisco (UCSF) criteria allowed patients with a single lesion of up to 6.5 cm, or two to three lesions of up to 4.5 cm, with a total tumour diameter (TTD) of a maximum of 8 cm and found that the rate of survival after LT was maintained [20]. When the explant tumour exceeded the UCSF criteria, the 5-year recurrence-free probability was 59.5% compared to 96.7% within the UCSF criteria. This model was also validated based on pre-operative imaging [21,22].

However, the use of morphometric variables only is considered to be suboptimal. Several groups combined surrogates of tumour biology, such as vascular invasion and grade of differentiation, into new composite selection criteria (Table 1). In extremis, the extended Toronto criteria excluded patients with poorly differentiated tumours upon biopsy of the largest lesion, cancer-related symptoms, extrahepatic disease or vascular invasion, regardless of the number or diameter of tumoral lesions. The 5-year cumulative risk of recurrence in the extended group was 30%, compared to 13% in the Milan group [23]. In a multicenter retrospective European study, Mazzaferro et al. proposed the up-to-seven criteria: the sum of the size of the largest tumour and the number of tumours for any given HCC could be “up to 7” if the microvascular invasion was absent. They reported a RR of 9.1% at 5 years for patients fulfilling the up-to-seven criteria [24].

Since the presence of microvascular invasion or the grade of differentiation is not available pre-operatively as standard when clinical decisions about prioritisation and listing need to be made, models using biomarkers, such as AFP and NLR, together with the diameter and number of HCC nodules, have been developed. In the Metroticket 2.0 model, the AFP value was incorporated in the up-to-seven criteria. Furthermore, AFP levels and the sum of the tumour diameter and number were significantly associated with HCC-specific death and this combination performed better than any other transplant criteria for HCC. To achieve 70% HCC-specific survival 5 years after LT in this study, the patients needed to have an AFP value below 200 ng/mL and a reported sum that could not exceed 7. For an AFP value between 200 and 400 ng/mL, the sum limit was up to 5 and, for a level of AFP between 400 and 1000 ng/mL, the sum was expected to be a maximum of 4 [25]. Another group combined a pre-LT AFP cut-off of 100 ng/mL together with the up-to-seven imaging criteria to define a low-and high-risk group for HCC recurrence risk stratification. The 5-year RRs were 9.4 and 44.5%, respectively [26]. The 5-5-500 rule (nodule diameter up to 5 cm, nodule number up to 5 and AFP value maximum of 500 ng/mL) resulted in a 5-year RR of 7.3% and identified patients at high risk of recurrence within the Milan criteria [27]. The combination of total tumour volume (TTV) below 115 cm^3^ and AFP below 400 ng/mL showed the predictive power of the post-transplant outcomes for candidates with HCC [28,29,30,31]. Patients within or beyond the Milan criteria but fulfilling the TTV/AFP criteria demonstrated comparable RR (4.5% vs. 9.4%) [30]. Sasaki et al. developed a pre-operatively assessable, continuous-risk score, Hazard Associated with Liver Transplantation for Hepatocellular Carcinoma (HALT-HCC), which associated the MELD-sodium, tumour burden score, AFP-value, year of LT, underlying cause of cirrhosis, NLR, locoregional therapy and Milan criteria status [32]. All the aforementioned models stress the importance of adding AFP to the scoring system, but the cut-off values they use are clearly heterogeneous.

The most promising model based on AFP was published in 2012 by Duvoux et al. and has been implemented as a set of selection criteria in France. In this AFP model, patients with one to three tumours, the largest of which has a diameter of 6 cm, or up to four lesions with a maximal diameter of 3 cm, are considered for LT if their AFP level is a maximum of 100 ng/mL. The AFP model had superior performance compared to the Milan criteria as the model identified a subgroup within the Milan criteria with a high risk of recurrence due to AFP values greater than 1000 ng/mL [33]. The model was shown in several validation studies to be highly predictive for the selection of patients with HCC beyond the Milan criteria [34,35,36,37,38]. In a review of 18 different risk scoring systems, the AFP model was the best-validated prediction model [39].

More recent recurrence-risk-assessment models have included the response to locoregional therapy prior to LT, as well as the evolution of AFP during treatment. A very useful tool is the online-accessible Metroticket calculator, which predicts the outcome of an individual patient with HCC considered for listing (expressed in 5-year survival and risk of HCC-related death after LT), starting from the radiology parameters and adapting the prediction to the variations in AFP and tumour morphology induced by locoregional therapy [25]. In 2020, in the first open-label, multicenter Italian RCT, the Metroticket calculator was applied to select patients beyond the Milan criteria with a 5-year estimated post-LT survival of at least 50% and used to assign them to LT or non-LT therapies, taking into account the radiological response and the evolution of the AFP value after downstaging [40].

Lai et al. combined the radiological response, AFP slope, NLR and length of waiting time (TRAIN score) as the selection criteria for the risk of intention-to-treat-death and recurrence. In the training cohort, the 5-year RR was 8.9% for the patients meeting the TRAIN score, 30% for those exceeding the score and, in the validation cohort, 13.8% and 100%, respectively [41]. Another model incorporated the AFP response (AFP-R), defined as the difference between the highest and final AFP-value before LT, into the New York/California (NYCA) score. An AFP-R consistently below 200 ng/mL predicted the best outcome. The score identified 85% of the patients beyond the Milan criteria with low or acceptable risk [42]. The model was externally validated and accurately predicted the RFS, with a 5-year RR of 9.5% in the low-risk category and 20.5% and 40.5% for the acceptable- and high-risk groups, respectively [43].

**Table 1:** Pre-transplant recurrence risk models.

Risk Model	Criteria	Recurrence Rate
Milan criteria [11]	Single tumour ≤ 5 cm	4-year RFS: 92.0%
	Or three tumours ≤ 3 cm	
	No vascular invasion	
UCSF criteria [21]	Single tumour ≤ 6.5 cm	5-year RFS: 96.7%
	Or three tumours ≤ 4.5 cm and TTD ≤ 8 cm	
Asan criteria [17]	Up to six nodules	3-year RR: 9.1%
	Largest nodule ≤ 5 cm	(beyond MC)
	No vascular invasion	
Up-to-seven criteria [24]	Sum size of largest lesion and number of lesions < 7	5-year RR: 9.1%
	No microvascular invasion	
TTV/AFP [30]	TTV < 115 m^3^	RR: 9.4%
	AFP < 400 ng/mL	(beyond MC)
5-5-500 model [27]	Number of lesions ≤ 5	5-year RR: 7.3%
	Size of lesions ≤ 5 cm	
	AFP ≤ 500 ng/mL	
Metroticket 2.0 model [25]	AFP < 200 ng/mL and size + number ≤ 7	5-year HCC specific survival: 70%
	AFP 200–400 ng/mL and size + number ≤ 5	
	AFP 401–1000 ng/mL and size + number ≤ 4	
AFP model [33]	Largest diameter (≤3; 3–6; >6 cm)	Low-risk: 5-year RR: 14.4% (beyond MC)
	Number nodules (1–3; ≥4)	
	AFP value (≤100; 100–1000, >1000 ng/mL)	
TRAIN score [41]	Response to locoregional therapies	5-year RR: 13.8%
	AFP slope cut-off 15 ng/mL/month	
	NLR cut-off 5	
	Length of waiting time (months)	
Toronto criteria [23]	No limits on size/number	5-year cumulative RR: 30% (beyond MC)
	Absent vascular invasion	
	Absent extrahepatic disease	
	Absent cancer-related symptoms	
	Biopsy not poorly differentiated	

NLR: neutrophil-to-lymphocyte ratio; RFS: recurrence-free survival; RR: recurrence rate; TTD: total tumour diameter; TTV: total tumour volume.

## 4. Risk Assessment Models to Predict Recurrence of HCC after Liver Transplantation

Post-operative recurrence risk assessment scores are often explant-based and include pathological parameters. Some pre-transplant scores can also be used in this setting to reassess the recurrence risk after LT. For example, the up-to-seven criteria originally took into account microvascular invasion [24]. The combination of several predictors of recurrence in UCSF patients in whom microvascular invasion was the strongest, in addition to tumour size above 3 cm, the presence of microsatellitosis and giant/bizarre cells involving more than 25% of the tumour, was used to stratify patients into clinically relevant risk groups [44]. The histopathological tumour features on the explants were also included in further models. Marsh et al. developed a model based on gender, tumour number, lobar distribution, tumour diameter and grade of vascular involvement [45]. Chan et al. identified similar independent significant explant findings that were predictive of recurrence: a tumour size of over 4.5 cm, macroinvasion, bilobar involvement and the grade of differentiation of the HCC [46]. Iwatsuki et al. combined bilobarity, the size of the largest tumour (2 to 5 cm and more than 5 cm) and the presence of vascular invasion (microscopic and macroscopic) to create a prognostic risk score with which to group patients into five grades of tumour-recurrence risk. This stratification correlated well with the RFS after LT (from grades 1 to 5: 100%, 61%, 40%, 5%, and 0% RFS at 5 years, respectively) [47]. Decaens et al. reported a novel scoring system that took into account tumour differentiation with higher accuracy than the Milan criteria. In the validation cohort, the 5-year TFS was 82.8% (low risk) and 50.0% (high risk) [48,49]. In a retrospective study, explant-based models were tested and the up-to-seven model provided the highest predictive accuracy of recurrence at 5 years, compared with the Decaens, Chan and Iwatsuki model [50]. Another retrospective study found that the Chan model had the highest value as a predictive model for 5-year recurrence compared to the Decaens and up-to-seven models [51]. These results indicate that in the post-transplant setting, there is also no consensus on the optimal model to use in clinical practice.

In addition to histopathological features, several new prognostic models include the AFP value to strengthen the risk stratification [52,53,54,55,56] (Table 2). The Risk Estimation of Tumour Recurrence After Transplant (RETREAT) scoring model identifies six levels of RR at 5 years using the AFP value determined before LT and adds three pathological features retrieved from the explant, namely microvascular invasion and the sum of the largest viable tumour diameter and the number of viable tumours. RETREAT performed better compared to the Milan criteria at predicting the risk of HCC recurrence. Furthermore, this score was validated in a large UNOS dataset. The researchers found an increasing risk of recurrence within 3 years from 1.6% in the lowest risk group to 29% for the group with a score of 5 or more; the time to recurrence also shortened as the RETREAT score increased. Additionally, the 3-year survival after LT was lower in the higher-risk groups (91% for a score of 0, 80% for a score of 3, and 58% for a score ≥ 5). [48,57]. However, the score was designed in a cohort who were mostly within the Milan criteria during their time on the waiting list. The score has been validated outside the USA in an European setting [58,59], but in this study, the prognosis of the patients after recurrence could not by differentiated with the RETREAT score [58]. Agopian et al. used these same clinicopathological variables combined with total cholesterol to develop a prognostic nomogram based on the data of 865 patients transplanted for HCC and reported the same excellent predictive value for recurrence after LT [55].

In 2017, the model of recurrence after liver transplantation (MORAL) score was proposed, using both morphological criteria and biological markers, including the NLR and AFP value. The author generated two different models from this dataset. Therefore, the MORAL score is applicable before and after LT or both. The pre-MORAL score (diameter of lesion more than 3 cm, NLR ≥ 5 and AFP-value > 200 ng/mL) resulted in RR values ranging from 20% to 100%. The post-MORAL score used four independent predictors of worse RFS available after LT (grade 4 differentiation, vascular invasion, size more than 3 cm and presence of more than three lesions). Both scores were superior to the Milan criteria at predicting the risk of recurrence [60]. In a multicenter study in Korea, the performance of the MORAL score was compared with various other LT criteria and proved to be the most strongly differentiating prognostic model for HCC recurrence in the setting of living-donor LT [61].

Most recently, Costentin et al. introduced the R3-AFP score as an assessment tool developed in a large international population, including a significant proportion of patients with HCC selected for LT with criteria expanded beyond the Milan criteria. The determinants included in the R3-AFP model are: the number of nodules, the diameter of the largest nodule, the presence of microvascular invasion, the nuclear grade and the last AFP value measured before LT. With this score, patients were stratified into four risk categories. In addition to the risk of recurrence at 5 years, the median time to recurrence and survival time after recurrence varied across the defined risk groups using the R3-AFP score. In the study, the new composite R3-AFP score was comparable to the RETREAT score in terms of performance [62].

**Table 2:** Post-transplant recurrence-risk models.

Risk Model	Criteria	Recurrence Risk
RETREAT [57,63] (post-LT)	Number of viable tumours + largest viable tumour diameter Microvascular invasion Last pre-LT AFP value (0–20; 21–99; 100–999; >1000 ng/mL)	3-year RR Score 0: 1.6% Score 1: 5.0% Score 2: 5.6% Score 3: 8.4% Score 4: 20.3% Score ≥ 5: 29.0%
MORAL [60] (pre-LT)	Size of nodules NLR Max. AFP value (>200 ng/mL)	5-year RFS Low-risk group: 98.6% Medium-risk group: 69.8% High-risk group: 55.8% Very-high-risk group: 17.9% (1 year)
MORAL [60] (post-LT)	Size of nodules Number of nodules Differentiation grade 4 Vascular invasion	5 year RFS Low-risk group: 97.4% Medium-risk group: 75.1% High-risk group: 49.9% Very-high-risk group: 22.1%
R3-AFP [62] (post-LT)	Number of nodules	5 year RR Very-low-risk group: 5.5% Low-risk group: 15.1% High-risk group: 39.1% Very-high-risk group: 73.9%
	Size of largest nodule Microvascular invasion Nuclear grade ≥ 2 Last pre-LT AFP value (≤100; 101–1000, >1000 ng/mL)	

AFP: alfa-foetoprotein, NLR: neutrophil-lymphocyte ratio, RR: recurrence rate, RFS: recurrence free survival.

## 5. Guidance for Surveillance of HCC Recurrence in the Setting of Transplantation

The working-group report from the ILTS Transplant Oncology Consensus Conference stressed that surveillance strategies after LT should be based on prediction tools to guide surveillance, but could not reach a clear consensus for clinical practice [64]. There are also no specific recommendations in the guidelines for the surveillance of HCC recurrence after LT [1]. A recent study in the USA showed that surveillance strategies remain highly heterogeneous, with 79% of the centers performing stratifications of transplant recipients for HCC recurrence risk, but in 19%, a specific protocol was missing. The majority of the centers had a routine imaging standard; however, a considerable heterogeneity related to the frequency and duration of HCC-recurrence surveillance was reported and the use of pre-LT AFP or specific cut-off values was variable [65]. Nevertheless, the surveillance of patients who underwent transplants for HCC proved to ameliorate survival. A recent multicenter study including 223 patients with recurrent HCC found that an increasing number of surveillance scans after LT (more specifically, three surveillance scans within the first 24 months) was associated with the timely administration of potentially curative treatment and improved post-recurrence survival [66].

The AASLD guidelines suggest surveillance with abdominal and chest CT every 6 months in the first 3 years after LT and repeated measurement of the AFP value in patients who present an elevated level before LT [67]. In the RETREAT study, the authors proposed HCC surveillance every 6 months for 2 years in patients with a score of 1 to 3 and the same interval for a longer time period of 5 years for those with a score of 4. Patients with a higher score should preferentially undergo HCC surveillance more frequently, every 3 to 4 months, for 2 years, followed by every 6 months for 2 further years. These patients are advised to undergo multiphasic abdominal CT or MRI imaging, chest CT and AFP measurement at the recommended intervals. Importantly, those with a RETREAT score of 0 did not receive surveillance [57,63]. In the UCSF group, the patients were followed with MRI scans at 3, 6 and 12 months and then underwent cross-sectional imaging every subsequent year. However more aggressive tumours (defined as poorly differentiated, beyond UCSF criteria or vascular invasion) on explants were examined frequently, every 3 months for 3 years and, subsequently every year. The AFP values were measured every 3 months in the first 2 years and then twice a year [60]. In the study by Costentin et al., there was no defined algorithm; in most of the centers, the monitoring consisted of 6-monthly CT or MR imaging and AFP measurement [62].

## 6. Conclusions

It remains challenging to individualise risk assessments for the recurrence of HCC after liver transplantation. This is increasingly important due to the rising number of patients transplanted outside the Milan criteria with extended criteria or locoregional therapy before or after listing for liver transplantation. There are different pre-and post-transplant recurrence-risk-assessment scores proposed in the literature that provide the possibility to stratify patients. However, there is currently no consensus on the preferred model. The most recent scores include biological markers for tumour behaviour or responses to therapy in addition to morphometric criteria. Further research is necessary to validate the discriminatory performance and clinical value of these scores. The systematic, routine use of recurrence risk assessment is advised to provide tailored advice for patient selection or prioritization and an adequate individualised surveillance strategy as this predicts the patients’ outcomes and prognosis. The optimal scoring system should be practical and provide a framework through which to design clinical trials adjusted to the risk of recurrence and test immunosuppressive strategies or new adjuvant therapies to prevent HCC recurrence after transplantation.

## Data Availability

Not applicable.

## Abbreviations

AFP	alfa-foetoprotein
CT	computed tomography
HCC	hepatocellular carcinoma
LT	liver transplantation
MELD	model for end-stage liver disease
MORAL	model of recurrence after liver transplantation
MRI	magnetic resonance imaging
NLR	neutrophil-to-lymphocyte ratio
RETREAT	risk estimation of tumour recurrence after transplant
RFS	recurrence free survival
RR	recurrence rate
TTD	total tumour diameter
TTV	total tumour volume
UCSF	University of California, San Francisco
